# Flooding, drowning, and health equity: The Lancet Regional Health – Western Pacific August editorial

**DOI:** 10.1016/j.lanwpc.2021.100264

**Published:** 2021-08-26

In mid-July, 2021, Zhengzhou, the provincial capital of Henan located in east-central China, was hit by record levels of rainfall. A year's worth of heavy rain beat down on the city over just 3 days. The rain washed away cars, swamped low-rise buildings, and swept through the city. Hundreds of people lost their lives in the flood; most of them were drowned.

Drowning happens throughout the year, but the increased access to water bodies for leisure activities and more heavy monsoon rainfalls in the warmer season put more people at risk of drowning. As the third leading cause of unintentional injury death globally, drowning kills around 235 000 people each year, and nearly two-thirds of these individuals are from the Asia-Pacific region. In the WHO *Regional Status Report on Drowning in the Western Pacific*, more than 74 000 deaths were attributed to drowning in the region in 2019. The incidence of drowning is disproportionally distributed across the region. More than 90% of drowning fatalities happen in natural bodies of water and even in domestic water storage containers in low-income areas. Among the deaths, children and adolescents living in rural areas are excessively affected.

The full scale of drowning in the Western Pacific region is obscured by many factors, such as poor data collection systems and low public awareness. Some drowning fatalities are never officially recorded or classified due to the remoteness of the incidents, and many non-fatal drownings are not documented as they should be. Additionally, drowning in climate-related extreme weather is not recorded or classified as related to the extreme weather in many countries. Nearly 50% of drowning deaths are not reported in countries most affected by natural disasters. In addition, local people might consider drowning to be a careless accident rather than a problem that can be systematically solved.

Evidence-based interventions have confirmed the effectiveness of formal swimming training and putting children under supervision in reducing childhood drowning. Other practical solutions include installing physical barriers to control access to water, training lifeguards, and establishing water safety and rescue teams. Additionally, imposing safe water transport regulations and improving disaster risk management have also been shown to reduce the incidence of drowning. The misconceptions surrounding drowning in many countries in the Western Pacific region is a result of inadequate resources and workforces to roll out drowning prevention strategies. While drowning is a more common issue in some areas of the region, other areas disproportionately benefit from drowning prevention programmes. Populations with the fewest resources are likely to be the least prepared to cope with the environmental threats. Apart from a small number of countries and areas in the Western Pacific region with well developed systems to prevent drowning, most areas of the region—especially those with the more severely affected populations—are still in the early stages of implementing small-scale water safety interventions at the local level.

The implementation of drowning prevention strategies is a multisectoral public health issue, which is related to different public health themes, including children's health, disaster risk management, transportation safety, built environment planning, and rural development, requiring the involvement from multiple stakeholders. As seen during the Zhengzhou flood, with the growing adverse effects of global climate change, more people in the region will be exposed to water-related disasters, and the risk of drowning will increase. Actions to prevent drowning need to be accelerated. Countries in the Western Pacific region need to coordinate efforts from different sectors to build up their national water safety plans and to develop and promote drowning prevention systems. These systems should be tailored to specific settings to reduce the avoidable deaths from drowning for the sustainable development of the region.

*The Lancet Regional Health – Western Pacific*

